# Theoretically framing views of people who smoke in understanding what might work to support smoking cessation in coastal communities: adapting the TIDieR checklist to qualitative analysis for complex intervention development

**DOI:** 10.1186/s12889-024-18923-x

**Published:** 2024-09-09

**Authors:** Emma Ward, Anna Varley, Melissa Wright, Ian Pope, Caitlin Notley

**Affiliations:** 1 Norwich Medical School, University of East Anglia, Norwich Research Park, Norwich, NR4 7TJ UK; 2 Patient and Participant Involvement (PPI) “expert by experience” representative, Great Yarmouth, UK

**Keywords:** Smoking, Smoking cessation, Intervention development, Qualitative, Health inequalities, Coastal communities, TIDieR checklist, E-Cigarette, Nicotine replacement therapy, Finanicial incentives, Behavioural support, Community intervention

## Abstract

**Introduction:**

People living in coastal communities have some of the worst health outcomes in the UK, driven in part by high smoking rates. Deprived coastal communities include socially disadvantaged groups that struggle to access traditional stop smoking services. The study aimed to seek the views of people who smoke living in coastal communities, to assess the optimal smoking cessation intervention for this population. In addition, the Template for Intervention Description Replication (TIDieR) checklist was adapted as an analytical framework for qualitative data to inform intervention design.

**Methods:**

Current or recent ex-smokers (*n* = 25) were recruited to participate in qualitative interviews from a range of community locations in a deprived English seaside town. A thematic analysis of the interview data was undertaken adapting the TIDieR framework. This analysis was triangulated with relevant literature and notes from stakeholder meetings and observations to map onto the TIDieR checklist to describe the optimal intervention.

**Results:**

Barriers to quitting smoking in the target population included low motivation to quit, high anxiety/boredom, normalisation of smoking and widespread illicit tobacco use. There was broad support for combining behavioural support, e-cigarettes and financial incentives, with a strong preference for the intervention to be delivered opportunistically and locally within (non-healthcare) community settings, in a non-pressurising manner, ideally by a community worker specially trained to give stop smoking support.

**Conclusions:**

An intensive community-based smoking cessation intervention was acceptable to the target population. Adapting the TIDieR checklist as a deductive qualitative analytical framework offered a systematic approach to intervention development. Combined with other intervention development activities, this ensured that the intervention design process was transparent and the proposed intervention was well defined. It is recommended that prior to intervention development researchers speak to members of the target population who may give valuable insight into the optimal intervention.

**Supplementary Information:**

The online version contains supplementary material available at 10.1186/s12889-024-18923-x.

## Background

The most effective smoking cessation aids are e-cigarettes [1], intensive behavioural support combined with pharmacotherapy [2], such as nicotine replacement therapy (NRT) [3] and financial incentives [4]. However, these evidence-based approaches to cessation have primarily been tested in motivated populations recruited to randomised controlled trials with strict eligibility criteria. Less is known about their effectiveness and applicability in real world community-based settings. We were unable to identify other studies which had sought views of people with lived experience of smoking on the potential for combining these elements in an intensive intervention. Intensive support may help to achieve the maximum possible cessation rate amongst those with most to gain from quitting smoking (e.g. an unemployed person with a chronic health condition who is a heavy smoker, suffering a double burden of both ill health exacerbated by tobacco smoking and financial hardship exacerbated by addiction) and represents good value for money given the cost effectiveness of effective smoking cessation interventions [5]. People living in deprived communities have some of the worst health outcomes in the UK, driven by extreme health inequalities [6]. One of the primary causes of these health inequalities is tobacco smoking [7]. An example of deprived communities is coastal areas where smoking rates are 6.7% higher compared to the rest of the UK [8]. The Chief Medical Officer, as part of a wider 2021 report into Health in Coastal Communities [8], recommended that the Government develop strategies targeting smoking in these areas. This may be necessary to meet the Government’s target for England to be smokefree by 2030 (defined as less than 5% smoking prevalence) [9].

‘Coastal communities’ are diverse populations, including socially disadvantaged households (e.g. low-income families, people unable to work due to illness/disability), older people with health-related problems, immigrant communities and other transient groups [8, 10]. Health inequalities in coastal communities are compounded by second-home ownership which impacts on housing affordability [11]. Groups living on low-income have specific needs meaning they struggle to access current stop smoking services (SSS) and have a lack of awareness of SSS or willingness to engage [12–14]. The Supporting Coastal Communities to Stop Smoking (SUCCESS) study set out to define a targeted intervention, potentially combining evidence-based components, acceptable to this population, with the ultimate aim of achieving high and sustained quit rates. To define this intervention effectively, substantial qualitative work needed to be undertaken in line with intervention development guidelines [15–17]. Coastal communities include ‘seldom heard’ groups, therefore it was essential to take an embedded approach, meeting community members in their locations and attempting to understand the realities of their lives. The aim of this study was to consult people resident in a deprived coastal community with lived experience of smoking about barriers to stopping smoking and their views on the feasibility and acceptability of potential intervention components. To maximise the usefulness of the exploratory qualitative data, a systematic analysis method was developed which could be mapped onto the existing standardised Template for Intervention Description and Replication (TIDieR) 12 item checklist typically used to describe interventions [18].

## Method

### Study Design

The SUCCESS study was undertaken in the east coast ‘seaside town’ of Great Yarmouth, which is one of the 20% of most deprived districts in England [19] with a smoking prevalence of 18% [20] (compared to the national English rate of 12.9% [21]). Ethical approval was sought from the UEA Faculty of Medicine and Health Ethics Committee to conduct audio-recorded semi-structured qualitative interviews with residents in a rural coastal community (FMH S-REC: ETH2223-0216). Community-based interview recruitment locations were purposefully selected to ensure that a range of people participated representing the different populations present within deprived coastal communities [8, 10]. Researchers monitored representation as data collection progressed and targeted community locations accordingly (for example, monitoring revealed that we hadn’t included young people on low incomes, so we approached a community college as one of our latter venues). The topic guide (supplementary material) used in these interviews was designed to explore contextual barriers and facilitators to stopping smoking for people living in a deprived coastal community and elicit participant perspectives on feasibility, acceptability and applicability of existing evidence-based approaches to quitting smoking.

### Participants and recruitment

Between October 2022 and March 2023, over 8 days in total, AV and EW visited community locations with permission from the managers to recruit and interview service users. These locations included two social supermarkets, two community cafés, a Portuguese café, a community college, a drop-in centre for the elderly, a drop-in centre for migrants, a women’s support centre and a men’s mental health support group. People were eligible to participate if they self-identified as being aged 18 years or above, were resident in the Great Yarmouth area, and smoked daily or had smoked daily but quit within the last 12 months. 22 current smokers and 3 ex-smokers gave informed consent before taking part in a confidential interview in-person at the community location on the day of the recruitment visit (23) or over the phone at a specified time following the visit (2). In-person interviews taking place in the community college were conducted in a private room. Other interviews were typically conducted in a communal space, after checking the participant was happy to proceed, with care taken to ensure the conversation was as private as possible (e.g. finding a quiet corner, undertaking interviews whilst other service users were busy with other activities). Interviews lasted approximately 30 min and participants were offered a £20 shopping voucher as a reimbursement for their time. One interview with a participant who did not speak English fluently was translated by a community worker.

### Measures and analysis

Demographic information and smoking/vaping status were collected verbally from participants by the researcher who later entered the information onto a secure spreadsheet. A framework analysis approach was undertaken [22] which involved EW and AV transcribing interview recordings (using Word 365 auto-transcription function, listening back to the recording and correcting mistakes) whilst making analytical memos and then uploading transcripts to NVivo qualitative analysis software platform [23]. The first eighteen interviews were coded inductively by EW and short summaries were written by EW and AV for each participant in a matrix using headings matching onto proposed intervention components. After reviewing the inductive coding and matrix, the research team decided to organise codes by adapting TIDieR items 2 to 8 and 11 to become overarching thematic headings because the headings fitted the data and allowed for feedback to be translated systematically. (TIDieR Items 1 and 9 were not relevant to analysis of the interview data and were therefore not included as theme headings, although Item 9 outlined in the proposed intervention description using the TIDieR checklist is informed by the interview analysis. TIDieR items 10 and 12 are specified by the TIDieR authors as not being relevant until the intervention study is complete, therefore they were not included as adapted qualitative theme headings.) After this analysis review, further participants were interviewed and their data were analysed using the adapted TIDieR coding framework. Saturation [24] (where no new themes were identified in analysis) was reached by the 25th participant. AV independently coded 10% of extracts using the TIDieR coding framework; coding was found to be consistent between both researchers. An interpretative analytical write-up was undertaken by EW using the TIDieR headings, prompting recommendations for intervention design. The analytical write up was shared with MW (SUCCESS PPI representative) who ‘sense checked’ the presented themes [25]. A consensus of theme validity was reached and is discussed in the findings section below.

### Additional intervention development work

In addition to the main qualitative study, following guidance on developing complex interventions [16, 17], additional work was undertaken including a literature search, meetings with borough council employees, and observations of smoking behaviours in the community. Findings from these activities were triangulated with the main qualitative analysis in a matrix using TIDieR item headings. The intention was for the additional intervention development activity to supplement the main qualitative study and demonstrate a systematic and transparent process from consultation with people with lived experience through to finalised design.

#### Literature review

The aim of the literature review was to identify existing smoking cessation interventions targeting UK coastal communities. A rapid review was undertaken searching publication databases [26–29] for relevant articles published within the last 20 years using key words (e.g., “coastal communities”, “smoking cessation”, “seaside”, “coast”, “smoking”). Due to a lack of relevant literature identified, the review was expanded to include systematic literature reviews of proposed intervention components and existing interventions similar to the proposed study targeting seldom heard populations.

#### Meetings with stakeholders

The aim of the meetings was to gather feedback on intervention ideas and explore potential ways of implementing the intervention within the local community. The meetings were informal and treated as patient and public involvement (PPI) work [25], designed to supplement the main qualitative study. Members of the research team met (1) a local stop smoking adviser; (2) two local borough council managers working with public health teams; and (3) two community workers delivering public health initiatives within the community. Meetings 1 and 2 took place online and meeting 3 took place in-person in the community. Meeting 1 took place before qualitative data collection and meetings 2 and 3 took place during qualitative data collection. Notes were taken during or after the meetings.

#### Non-participant observations

The aim of the observations was to understand the smoking environment of the area and how it might impact future intervention delivery. Observations of smoking behaviour and environment (e.g. proliferation of vape shops, shops selling illicit tobacco, evidence of cigarette butts) were undertaken in the town centre and at the community locations used for interview recruitment. Brief notes were taken.

## Findings

The subsections below with TIDieR headings report the qualitative interview data analysis. Triangulation with the additional intervention development work is reported in a later subsection. Table 1 shows the characteristics of the 25 interview participants. Participant codes used to reference quotes refer to a participant’s gender, age, and smoking/vaping status (e.g. ‘M58_S’ for ‘male aged 58 who only smokes’). Some quotes have been edited to improve readability by removing repeated/redundant words and discourse markers (e.g., ‘um’, ‘er’).

**Table 1:** Profile of participant characteristics (*n* = 25)

	Sample
Gender: Male Female	48% (12) 52% (13)
Age: Range (years) Mean (years)	18–84 46.9 (SD 16.588)
Ethnicity: White British Black British White Portuguese White Romanian	84% (21) 4% (1) 8% (2) 4% (1)
Occupation: Employed Self-employed Unemployed or long-term sick Carer Student Retired	16% (4) 4% (1) 40% (10) 8% (2) 12% (3) 20% (5)
Indices of Deprivation [36] 1 (10% most deprived) to 10 (10% least deprived) Missing *n* = 2 1 2 3 7	65.2% (15) 21.7% (5) 8.7% (2) 4.3% (1)
Smoking/vaping status: Smoking only Dual using e-cigarettes and tobacco Vaping only	84% (21) 4% (1) 12% (3)

Table 2 shows the original TIDieR checklist item and the checklist item adapted into qualitative overarching themes and the themes and subthemes within each item heading derived from analysis.

**Table 2:** TIDieR Items and adapted qualitative themes headings with an overview of the analysis displaying themes and subthemes derived from analysis

Original TIDieR Item	Adapted qual theme heading and description (in italics)	Themes and subthemes
**Item 2 Why**: Rationale, theory, or goal of the elements essential to the intervention	Why? Perspectives on contextual factors that demonstrate a need for intervention and areas to target *The item was adapted to focus on exploring data related to the rationale for the need for an intervention generally and areas to target to overcome perceived contextual barriers to smoking cessation.*	• Motivation: o Lack of intrinsic motivation to quit o Health misinformation about smoking o Specific motivational factors (health, pregnancy, family, cost, fitness) • Emotion management: o Stress or anxiety o Boredom or loneliness o Identity, routines and enjoyment/pleasure • Smoking normalisation: o Smoking visibility and prevalence in community o Intergenerational and peer smoking o Smoking functioning to solidify bonds/foster relationships o Illicit tobacco availability and use • Community meanings: o Lack of engagement with wider community o Unsafe communities o ‘Empty’ communities o Micro communities (family/friends/community groups)
**Items 3&4 What**: Physical or informational materials (3) and procedures, activities and processes (4) used in the intervention	What? Perspectives on potential intervention component materials and procedures *These items were adapted to focus on exploring data related to general ideas for potential intervention components. These included interventions which had previously been shown as effective in different populations, and included provision of an e-cigarette or NRT, behavioural support for smoking cessation, and financial incentives for stopping smoking.*	• Provision of e-cigarettes or NRT: o Experiences of e-cigarettes and NRT o Beliefs about e-cigarettes o Motivation to try e-cigarette as part of proposed intervention o E-cigarette device type and flavour preferences o Provision preferences (starter kit vs. vape shop vouchers) • Behavioural support: o Experience of behavioural support o Motivation to engage in behavioural support as part of proposed intervention o Preference for non-judgemental/non-pressurising delivery style • Financial incentives: o Financial motivations to stop smoking o Views on effectiveness of financial incentive as part of proposed intervention o Ethical issues (morality of funding payments; potential for payment to be used for cigarettes) o Perspectives on incentive format (community-based vs. cash payment vs. vouchers)
**Item 5 Who provided**: Expertise, background and specific training of intervention provider	Who provides? Perspectives on expertise, background and specific training of person providing the intervention *This item was adapted to focus on exploring data related to general ideas about who could deliver the intervention and why they were appropriate.*	• Experiences of professionals delivering smoking cessation support • Community worker preference (over healthcare professional) for proposed intervention • Reassured by healthcare knowledge/background
**Item 6 How**: Modes of delivery of intervention	How? Perspectives on the mode of delivery of the intervention *This item was adapted to focus on exploring data related to participants’ perspectives on different modes of delivery generally.*	• Barriers to accessing smoking cessation support (work, cost, physical and mental health) • Opportunistic delivery preference • Flexibility necessary for follow up mode of delivery
**Item 7 Where**: Types of locations where the intervention occurs, including any necessary infrastructure or relevant features	Where? Perspectives on where the location the intervention should be delivered *This item was adapted to focus on exploring data related to participants’ perspectives on potential locations to deliver the intervention.*	• Perspectives on publicity locations • Perspectives on locations to deliver proposed intervention (community groups, workplaces, pharmacies, medical centres) • Perspectives on follow up locations
**Item 8 When and how much**: Number of times the intervention was delivered and over what period of time including the number of sessions, their schedule, and their duration, intensity or dose	When and how much? Perspectives on the number of intervention timings, duration and intensity *This item was adapted to focus on exploring data related to participants’ perspectives on potential timings, duration, and intensity.*	• Perspectives on financial incentive amount • Perspectives on follow up frequency • Perspectives on combining components
**Item 11 How well**: How and by whom intervention adherence or fidelity was assessed	How well? Perspectives on evaluating the intervention *This item was adapted to focus on exploring data related to participants’ perspectives on acceptability of different research methods used within the evaluation of the intervention.*	• Perspectives on community and individual randomisation • Perspectives on acceptability of monitoring (CO testing vs. urine sample) • Exploitation of monitoring

### Item 2: Why? Perspectives on contextual factors that demonstrate a need for intervention and areas to target

The interview data revealed that smoking was normalised within the communities, with participants describing a visible smoking prevalence on the streets, intergenerational and peer smoking, and widespread illicit tobacco use making smoking more affordable. Participants described smoking as a simple pleasure allowing fleeting relief from lives that were often experienced as stressful, boring, or lonely:

*‘When I’ve been to the doctors and they say if “I don’t give up smoking, I’ve only got so many years this and that”, I think to myself, well, I’ve got nothing else in my life. I’ve got no like family, I’ve got nothing, it doesn’t matter.’ (M58_S)*

Anxiety was a common theme throughout the interviews and smoking was seen as a coping mechanism, always available in times of need. It was the main reason given for relapse. Some participants described anti-social behaviour in their neighbourhoods and commented that they felt unsafe. Although participants appreciated the ‘sea air’ and the beach, Great Yarmouth was generally perceived as a town in decline with vacant shops and a lack of facilities. Within this ‘unsafe’ and ‘empty’ community context, smoking was reportedly used by participants as a tool to solidify bonds and foster relationships with friends and family:

*‘Smoking is a social thing. Me and my neighbour are always, “what are you doing?” “Nothing”. “Shall we have a coffee and a fag then?” And then we’ll stand at the front [of house] having a cigarette.’ (F42_S)*

Motivation to quit was low amongst the participants, with some not wanting to give up the enjoyment they received from smoking, seeing it as an integral part of their identity. Some did not believe quitting would improve their health. Others knew that they should give up to improve their health, but lacked self-efficacy and reported that they would only be motivated if faced with a serious health scare. Those who had managed to quit, however, at least for a short while, stated that they had done so primarily due to a desire to improve fitness, for their family, pregnancy, or to save money:

*“It was just getting too expensive.” (F44_V)*

### Items 3 & 4: What? Perspectives on potential intervention components

#### Provision of e-cigarette or NRT

Most participants (17) had tried and failed with NRT in the past and no longer perceived it to be a viable option to help them to quit smoking. Participants had varied experiences of vaping ranging from managing to swap completely from tobacco, through to reporting being too intimidated or uninterested to try vaping. One participant was dual using citing being able to vape in places they couldn’t smoke as their main motivation to vape. Some found vaping to be more expensive than the illicit tobacco they used; others had bought illicit disposable vapes capable of thousands of puffs making vaping the more affordable option. Participants who had tried vaping but had not managed to switch, discussed finding vaping unsatisfying compared to cigarettes. In addition, many participants had reservations about e-cigarettes and were disbelieving or suspicious of public health messages supporting their use for smoking cessation. Concerns raised included potential unknown long-term harms, “popcorn lung”, e-cigarette or vape use-associated lung injury (EVALI), addictiveness, and potential fire risks:

*‘I see a video saying this bloke had one and he went to fill it up and it blew up his face. That put me right off.’ (M51_S)*

Despite these mixed past vaping experiences and reservations about e-cigarettes, nearly all participants stated that they would accept and try an e-cigarette if they were offered one as part of the intervention, even though some were dubious about their chances of success. Being offered the e-cigarette meant that no financial investment was needed on behalf of the participants, which had put off some from trying vaping in the past. Provision of an e-cigarette for free, regardless of participants’ intention to quit, was a welcomed low-pressure approach; participants commented that as they had ‘nothing to lose’ they ‘might as well’ try vaping:

*‘I would try it to see if I would like it or not like it. If it’s going to help me quit cigarettes I will try it.’ (M42_Sa)*

Most participants wanted to be given a starter kit rather than a vape shop voucher. Travelling to a vape shop was perceived as a barrier by some, because they were busy or would struggle to afford the bus fare. Some participants described being intimidated by vape shops and feeling that they would be more reassured about the e-cigarette’s safety and effectiveness if it was given to them as part of the intervention:

*‘It can be quite overwhelming walking in to a vape shop. I remember when I bought my first vape, it was awful, I just didn’t know where to start. Especially if you’re given a voucher and you have to stick to that amount, it’s really hard to do that. A starter kit would be a good idea.’ (F28_V)*

Participants who had found success with vaping reported enjoying experimenting with flavours. Other participants commented that they were put off e-cigarettes because they didn’t replicate the taste of cigarettes, perceiving flavours to be ‘silly’. Those who had tried vaping unsuccessfully in the past described issues with functionality such as devices leaking or breaking, whereas participants currently vaping preferred disposable e-cigarettes (sometimes larger illicit models) because they were easy to use, tasted nice and were convenient. Therefore, they believed that intervention participants should be offered a similarly simple device:

*“Easy to use. I haven’t got to fiddle about cleaning and changing the coils”, (F51_S)*

#### Behavioural support

Participants had experienced a range of behavioural support for smoking cessation in the past ranging from no support through to engaging fully with the local Stop Smoking Service (SSS). Those with SSS experience reported mixed experiences; a couple had found it very useful, describing attentive advisors who took time to listen, whereas a couple described minimal or impersonal interactions and cancelled appointments. Some experienced seeing their CO reading reduce overtime as motivating. Behavioural support was generally viewed as being of little help however, with participants believing that quitting could only be achieved alone with high intrinsic motivation or that engaging with behavioural support would be too pressurising. Participants felt that any behavioural support delivered as part of the intervention would have to be delivered non-judgementally, empowering people to make their own choices through building positive accepting relationships:

*‘I think pressure would be the main thing that would put people off. If we can have a conversation and be like friends about it then yeah I think that’d be better[…] If someone stands preaching at you about smoking’s bad for you, no-one’s gonna listen.’ (M18_S)*

#### Financial incentive

The idea was acceptable to nearly all the participants, although a couple felt that it was unethical to use public money to financially incentivise people to stop smoking and that quitters should be intrinsically motivated. There were mixed views on whether it would be successful in motivating people to quit with some stating that it would be very motivating; some stating that it would be a nice ‘added extra’, reinforcing their quit but not their main motivation; and others believing that it would have no impact because they were not intrinsically motivated to quit. Levels of deprivation and cost-of-living were reasons given by some about why they thought it might work in their area specifically:

*‘Money’s a great motivator in Yarmouth, because there’s so little of it. You’re onto a winner with that. I think a lot of people would be motivated by the thought of money if they quit.’ (M59_S)*

Participants liked idea of a community incentive for stopping smoking, with money paid to a local charity or community project following a successful quit, but most felt that community incentives wouldn’t motivate people from the area. Instead, participants felt personal payments would be more motivating. Some preferred cash as it was more convenient, although others pointed out that vouchers might be the better option to prevent people spending money on cigarettes:

*‘I would rather have it in vouchers because then I know I’d spend it on food in [supermarket]. If I got money, I may end up spending it on things that I shouldn’t spend it on.’ (F52_Sb)*

### Item 5: Who provides? Perspectives on who could deliver the intervention

Interactions about smoking with healthcare professionals (HCP), such as GPs or practice nurses, had generally been experienced as negative by participants, describing HCPs as either being condescending or seeming disinterested or apathetic. A few participants gave examples of requests for help with quitting not being followed up by HCPs. These experiences influenced participants’ preference for the intervention to be delivered by a community worker with existing relationships within the area, although a couple commented they would be more reassured if the advisor had a healthcare background. Participants believed that being familiar with the issues participants faced would mean the people giving advice were willing to spend time and be more understanding:

*‘Probably people in the community [should deliver the intervention]. People who are face to face with local residents because they know who they are, they trust them and they’d be more likely to listen to them.’ (M43_S)*

### Item 6: How? Perspectives on the mode of delivery

Participants discussed practical barriers to accessing healthcare such as being too busy working or not being able to get to appointments due to cost or other issues such as mental health. Most commonly, however, participants lacked quitting motivation and, due to fear of failure, did not want to initiate a formal quit attempt by seeking support. Most participants discussed that if support was offered opportunistically face-to-face ‘there and then’, in a non-pressuring manner, they would take it up as it required minimum practical or emotional investment:

*‘People struggle to get into the doctors as it is, let alone…so maybe more this sort of thing [mental health drop-in café][…], something a bit more human. (M40_S)*

*Yeah, I think that’s [opportunistic delivery] a brilliant idea. People need to be encouraged, but in the right approach. I think if you’re coming too strong. It pushes you the other way.’ (M52_S)*

Regarding following participants up to offer continued support and incentives, there was not a standout mode of delivery proposed by participants. Some preferred remote methods and others preferred face-to-face. The same flexibility was discussed regarding provision of ongoing e-liquid supplies with a couple commenting that receiving them in the post would be a preferred option. Personal preferences hinged on what method was perceived to be the most convenient and least anxiety provoking:

*‘[I’d prefer] one-to-one because I’m a bit wary of new people and stuff like that as well. I’ve got learning disabilities and depression, when I can’t communicate properly I feel frustrated.’ (M38_S)*

### Item 7: Where? Perspectives on where the intervention should be delivered

Most participants commented that the opportunistic intervention delivery should take place in the community, using locations such as community groups, food banks, libraries, and cafes:

*‘They could come to [social supermarket] where I work and then there’s the church where the foodbank is, I think somewhere like that, and there’s a café on our high street, a mental wellbeing café. I think somewhere like that cause a doctors might be intimidating for people… It needs to be really local.’ (F52_Sa)*

Some participants thought that focusing solely on community locations open during the day might mean that the intervention would not reach younger or employed people who smoke as they were less likely to use those services. They suggested also targeting pharmacies, colleges and workplaces:

*‘I have a lot to do in my life […] It would be helpful if you came to see me at my work’. (M42_Sb)*

Participants suggested follow up support to be delivered either in the community, at home or remotely, with preferences dictated by what was personally perceived as most accessible and least anxiety provoking.

### Item 8: When and how much? Perspectives on intervention timings, duration, and intensity

Regular follow-up meetings to offer ongoing support and incentives were generally acceptable to participants, if, as described above, appointment times/locations were flexible and convenient. Monthly meetings were considered to be manageable and not too intrusive. £20 payments for clear testing, followed by a bigger payment at final follow-up was acceptable to those who felt that they would be motivated (at least in part) by financial incentives:

*‘I think you’d probably get people snatching your hand off for £20 a month because that’s probably a week’s worth of gas and electric for some people.’ (F44_V)*

There were a few participants who wanted to continue smoking and believed that no intervention (including financial incentive amount) would motivate them to quit. However, most of the participants believed that combining components could work and promote engagement:

*“I think [combining approaches] would be the best because then you have support, you have e-cigarettes to help you cut down and then there’s more motivation with money.” (M18_DU)*.

### Item 11: How well? Perspectives on evaluating the intervention

Providing a clear CO reading to qualify for an incentive and assess the effectiveness of the intervention was more acceptable to the participants than providing a clear urine sample. Some participants were already familiar with CO testing through SSS support they had received in the past. Participants had concerns that those who hadn’t managed to stop smoking would exploit urine testing by providing fake samples using non-smokers’ urine instead of their own. In addition, a few felt the urine testing was too personal or was stigmatising:

*‘[Doing a urine test] is intimidating, almost like a drug addict, or you’ve come out of prison and have to see your parole officer.’ (F65_S)*

Participants who commented did not like the idea of a randomised controlled trial (RCT), either on an individual randomisation basis or a cluster randomisation approach within different communities. It felt unfair to the participants, and they thought that having a chance of not receiving support could put some smokers off:

*‘I don’t think [randomisation] is fair. I certainly wouldn’t take part. If I wanted to stop smoking I would want to help given to me, not told that I might get it’. (F52_Sa)*

### Triangulation with additional intervention development work

Findings from the main qualitative study and additional development work were summarised and triangulated using a matrix to formulate recommendations for the intervention design. This is presented in Table 3 and summarised in this section.

**Table 3:** Summary of qualitative interview findings triangulated with summary of findings from additional intervention development work with resulting recommendations for intervention design

Item no.	Item	Interviews	Stakeholder meetings	Observations	Literature	Recommendations for intervention design:
1	BRIEF NAME	SUCCESS: Supporting Coastal Communities to Stop Smoking				
2	WHY Describe any rationale, theory, or goal of the elements essential to the intervention	Multiple barriers to quitting smoking described including low quitting motivation and confidence, lack of support, exposure to other smokers at home and in community, smoking culture, mistrust and lack of understanding of smoking cessation support.	SSS advisor identified multiple barriers including illicit tobacco, widespread poor mental health, ageing smoking retirees and immigrant communities with embedded smoking culture, lack of willingness to engage in services until health impacted.	Smoking behaviour and evidence in community very visible and widespread. Illicit tobacco openly sold.	High rates of smoking in coastal communities^8^. Multiple barriers to quit smoking including mental health issues, cultural norms and lack of willingness to engage with support^12–14^. Lack of existing interventions targeting this group.	- Innovative and ambitious approach to smoking cessation needed to support people who smoke living in coastal communities to quit to overcome multiple barriers to smoking cessation.
3 & 4	WHAT Materials: Describe any physical or informational materials used in the intervention, including those provided to participants or used in intervention delivery or in training of intervention providers. Provide information on where the materials can be accessed (e.g., online appendix, URL). Procedures: Describe each of the procedures, activities, and/or processes used in the intervention, including any enabling or support activities.	E-cigarettes were more attractive to participants than NRT. This was due to past failures with NRT and scepticism about its efficacy. Views on vaping were mixed, with a couple successfully transitioning from tobacco but others finding it unsatisfying or facing barriers such as cost, feeling intimidated in vape shops or concerned about vaping safety. Despite these reservations, most participants were open to trying e-cigs if offered as part of an intervention, especially if provided for free. Behavioural support experiences varied, with some finding it helpful in the past, especially the CO monitoring. However, lack of motivation and fear of failure deterred participants from seeking formal smoking cessation support. They had a preference for behavioural support with minimal pressure. Financial incentives were generally accepted, though opinions on their effectiveness varied. Participants favoured personal payments over community incentives, with vouchers seen as a practical way to deter spending on tobacco.	SSS advisor has experience of delivering effective e-cig support scheme in GY. Giving quitter e-cig worked better than vape shop vouchers as it offered instant support and consistency. Essential to offer ongoing supplies to ensure engagement and combat access to cheap illicit tobacco. SSS stated that people needed educating on relative risks of vaping compared to tobacco as many people have negative risk perceptions of vaping and don’t understand the differences in nicotine delivery mechanisms. SSS advisor believed that incentives would be attractive to residents due to cost of living. SSS had found that a low pressure approach worked better when conducting community outreach.	Proliferation of vape specialist shops in community allowing easy access to legitimate vaping products. Deprived area and people on low incomes indicating that financial incentives would benefit residents. Illicit vapes being sold in some shops.	E-cigs more effective than NRT^1^. E-cigs feasible as intervention for people from lower socio-economic backgrounds^33,34,35^. E-cig perception barriers for implementation^35^.	- Offer an e-cigarette and supplies for no cost (rather than NRT) - Provide a starter kit directly to participants rather than a vape shop voucher - Ensure the device is simple to use and cheap to run - Offer a range of flavours including tobacco - Offer ongoing supplies of e-liquid - Incorporate CO monitoring into behavioural support - Offer a financial incentive alongside other intervention components - Provide individual level incentives - Provide vouchers for clear CO readings - Reassurance that participants do not have to intend to quit smoking to take part - Advisors focus on building rapport with participants, keeping interaction light and non-committal to avoid the participant feeling pressurised - Advisors use language sensitively (e.g. avoiding talking about quitting and instead focus on switching, encouraging participants to ‘just try it’ as they have ‘nothing to lose’) - Advisors should discuss how people generally are more likely to switch away from smoking with support - Emphasise to the participant that the device has been specially selected for its usability and effectiveness - Discuss (non-smoking cessation) motivations to vape (e.g. vaping in places smoking not allowed, cut down, saving money etc.). - Discuss how vaping and smoking are different in terms of nicotine delivery and usage patterns to address addictiveness/effectiveness concerns - Combat e-cigarette myths in a sensitive way acknowledging people’s anxieties - If appropriate, inform the participant about the dangers of illicit e-cigarettes
5	WHO PROVIDED For each category of intervention provider (e.g., psychologist, nursing assistant), describe their expertise, background and any specific training given.	Participants had generally negative experiences discussing smoking with healthcare professionals, feeling they were often condescending or disinterested. They stated they preferred community workers to deliver the intervention, desiring advisors familiar with their issues and could devote time to support them. Some stated that advisors with healthcare backgrounds would reassure them about the support coming from someone other than a healthcare professional.	Meeting with borough council managers revealed community workers delivering other public health support such as screening and assistance in accessing appointments: smoking cessation support would also be within remit. Discussion with community workers showed a willingness to be involved if sufficient training was provided.		Non-healthcare professionals have been trained to deliver opportunistic smoking cessation interventions^34^.	- Community worker delivers intervention - Community worker undertakes HCP smoking cessation training - Community worker informs participants that they have received HCP smoking cessation training to reassure them of credentials
6	HOW Describe the modes of delivery (e.g., face-to-face or by some other mechanism, such as internet or telephone) of the intervention and whether it was provided individually or in a group.	Participants highlighted barriers like time constraints, cost of transport, and mental health hindering their ability to access healthcare. They favoured opportunistic, face-to-face support. Preferences for follow-up and e-liquid supply delivery varied, emphasizing convenience and methods that would cause the least anxiety.	SSS advisor stated that people were often anxious about engaging with them following a referral from a healthcare professional. Community outreach had worked well in the past but SSS capacity was limited to undertake this work. Community workers could be well placed to deliver to support as similar modes of delivery were being implemented for other health behaviours		Opportunistic delivery of smoking cessation interventions proven feasible^33,34^	- Offer the intervention to participants opportunistically at point of recruitment - Advisors should take a flexible approach to follow up mode of delivery, following the participant’s preferences
7	WHERE Describe the type(s) of location(s) where the intervention occurred, including any necessary infrastructure or relevant features.	Participants preferred opportunistic intervention delivery in community settings like food banks, libraries, and cafes. Targeting pharmacies, colleges, and workplaces for broader reach was also suggested. Follow-up support could occur in community, at home, or remotely, based on accessibility and comfort levels, ensuring inclusivity for a range of people.	Community workers could identify existing clients to target as well as a knowledge of local community locations and existing network connections. They did not anticipate recruitment to be problematic. Community workers already working flexibly to meet their clients needs.	High proportion of smokers accessing community organisations. Community organisations supportive of proposed study.	Opportunistic delivery has been successfully delivered in locations such as healthcare settings^13,33^, homeless centres^34^, places of worship^13^ and community centres^13^.	- Advisors should proactively recruit people from a variety of community locations to ensure a diverse range of people who smoke are reached - Advisors should take a flexible approach to follow up location, following the participant’s preferences
8	WHEN and HOW MUCH Describe the number of times the intervention was delivered and over what period of time including the number of sessions, their schedule, and their duration, intensity or dose.	Participants stated that regular follow-up meetings for support and incentives would be acceptable if flexible. Monthly meetings were considered manageable. Financial incentives, like £20 for clear testing and a larger final payment, would motivate some. While did not desire quiting regardless of intervention, most believed in the effectiveness of combined interventions and expressed willingness to engage.	SSS advisor felt that combining components could be effective in overcoming multiple barriers. Monthly meetings were felt manageable by community managers and workers.		No studies combining components – efficacy and cost effectiveness remain untested. Suggested incentive amounts within range typical for financial incentives studies^4^.	- Offer follow up support at monthly intervals post recruitment. - Follow up support sessions will occur at a flexible time and location - Offer voucher incentives including bigger payment at the final monthly appointment. - Combine components for maximum take-up and impact
9	TAILORING If the intervention was planned to be personalised, titrated or adapted, then describe what, why, when, and how.	Barriers to quitting included attitudes and beliefs participants held about smoking function, smoking identity and normalisation, motivation to quit, health impacts of smoking, and financial impacts.	SSS said that some people are motivated by learning about the impact of smoking on loved ones (including pets) and impact to undertake physical activity.			Behavioural support should be tailored depending on the information given by participant, potentially covering: - The function smoking plays in participant’s life, identifying triggers for relapse prevention (e.g. to relieve stress, anxiety, and boredom). - Smoking identity and the normalisation of smoking within participant’s life (e.g. family/friends/household smoking) - Beliefs about smoking’s impact on health - combat misinformation in a sensitive way - Combat prevailing belief that high intrinsic motivation is essential to quit (e.g. give examples of ‘accidental quitters’, by using no pressure language, informing more likely to succeed with help etc.) - Explore other potential motivators than health (e.g. cost, starting a family, protecting existing family and pets, personal fitness) - If appropriate, inform the participant about the dangers of illicit tobacco
10	MODIFICATIONS	n/a	n/a	n/a	n/a	n/a
11	HOW WELL Planned: If intervention adherence or fidelity was assessed, describe how and by whom, and if any strategies were used to maintain or improve fidelity, describe them.	Participants preferred CO testing over urine sampling for incentives and measuring intervention outcomes. Some had positive prior experience with CO testing from SSS. Concerns arose regarding the potential for exploitation and privacy issues with urine testing. Participants disliked the idea of randomized controlled trials, fearing unfairness and that this would deter people from participating.	SSS advisor believed that CO reading would be better than urine because people would be suspicious about the purpose of a urine test.	Community groups willing to be involved in future intervention.	Cluster randomisation undertaken in an opportunistic study for ethical and practical reasons^34^. CO validation most common method in smoking studies^1^.	- Use CO testing (rather than urine sample) to validate quits at follow ups - Avoid individual randomisation
12	HOW WELL	n/a	n/a	n/a	n/a	n/a

Consultation with a stop smoking advisor (SSS) working in Great Yarmouth, and non-participant observations, supported the interviews and literature [8, 14] highlighting the multiple barriers to quitting smoking for those living in coastal communities. The SSS advisor we consulted described how their own provision of a starter kit to (motivated) Great Yarmouth quitters had been popular and effective, supporting evidence of similar schemes [30, 31]. Another recommendation generated by the interview data had been to offer e-cigarettes rather than NRT, also supported in the literature showing e-cigarettes to be more effective than NRT [2], and feasible in other opportunistic interventions targeting people from lower socio-economic backgrounds [32–34].

Qualitative feedback we gathered strongly supported an approach which brings the intervention to smokers in the community, delivered in an opportunistic manner, rather than expecting smokers to be motivated or confident enough to approach health services (also evidenced in the literature [13]). Discussions with the local stop smoking service indicated that although they sometimes undertook successful outreach, their capacity to fully engage with this type of work was limited. Meetings with the borough council revealed that there are community workers employed by the local authority already undertaking community work around health and wellbeing (e.g. diabetes screening) and that their role could be potentially adapted to incorporate smoking advice. Researchers spoke to two such community workers who were supportive of the idea and believed that they would be able to recruit to the study. The community groups visited during observations were also supportive of the intervention and commented that they would agree to delivery taking place on their premises. These discussions with other stakeholders illustrated the need to ensure the impact of context surrounding intervention delivery is evaluated when the intervention is implemented.

## Discussion

In this study we set out to qualitatively explore the views of people who smoke living in coastal communities, to assess the optimal smoking cessation intervention for this population. Using interview data we demonstrated how the TIDieR checklist headings could be adapted and used as deductive themes (Table 2). In line with MRC guidance on developing complex interventions [16, 17], the qualitative analysis was triangulated with current relevant literature and other intervention activity including meetings with other stakeholders and non-participant observations, by mapping findings onto the TIDieR checklist and informing recommendations (Table 3).

Reducing smoking in coastal communities is seen as a policy priority to reduce health inequalities [8]. Our findings demonstrated that this is likely to be challenging due to the normalisation of tobacco use within coastal communities, widely held beliefs about willpower alone being sufficient for success, and complex attitudes towards smoking cessation support options. Innovative approaches to support smoking cessation targeting these communities are clearly needed. Following triangulation of the data sources, the research team agreed that it is highly likely that incorporating e-cigarettes and incentives would be acceptable to the population, if delivered opportunistically in (non-healthcare) community settings, ideally by a community worker. The TIDieR description of the finalised SUCCESS intervention is outlined in Table 4 (and incorporated into the study logic model, see supplementary information).

**Table 4:** The TIDieR (Template for Intervention Description and Replication) Checklist completed for the SUCCESS study

Item no.	Item	Intervention description
1	BRIEF NAME Provide the name or a phrase that describes the intervention.	Supporting Coastal Communities to Stop Smoking (SUCCESS)
2	WHY Describe any rationale, theory, or goal of the elements essential to the intervention	SUCCESS was developed using the COM-B theory of behaviour change and aims to support people from coastal communities to stop smoking (including smokers who are not motivated or intend to quit smoking). Coastal communities are deprived areas with health inequalities driven in part by high smoking prevalence. The intervention combines evidence-based approaches (provision of an e-cigarette, behavioural support, financial incentive) via the application of evidence-based behaviour change techniques (BCTs).
3	WHAT Materials: Describe any physical or informational materials used in the intervention, including those provided to participants or used in intervention delivery or in training of intervention providers. Provide information on where the materials can be accessed (e.g., online appendix, URL).	The SUCCESS programme includes **provision of an e-cigarette starter kit** and eliquid for no cost with instruction from an advisor on how to use it. A range of eliquid flavours will be offered to participants. Participants will be supplied eliquid at follow up support sessions to reduce financial burden on the participants and encourage continued adherence. Participants will be offered a **financial incentive following a clear CO test** for stopping smoking in the form of voucher payment at each follow up support session. Advisors will be trained using **bespoke training** developed by the research team (who are also trained smoking cessation advisors) specifically for the intervention. This training will be designed to be brief and accessible. Training materials and intervention handbook will be made available online through an open access platform.
4	WHAT Procedures: Describe each of the procedures, activities, and/or processes used in the intervention, including any enabling or support activities.	The SUCCESS programme includes **brief advice** delivered at recruitment and at follow up support sessions in the form of a behavioural support conversation. Factors that could influence smoking behaviour will be discussed such as smoking beliefs, motivations, and goals. The brief advice also includes discussion relating to factors that could influence vaping behaviour such as e-cigarette beliefs and motivations and how vaping differs from smoking. The advisor training will cover tone of intervention delivery as well as intervention content, training advisors to deliver the intervention in a non-judgemental, non-pressurising and sensitive manner.
5	WHO PROVIDED For each category of intervention provider (e.g., psychologist, nursing assistant), describe their expertise, background and any specific training given.	SUCCESS will be delivered by c**ommunity workers**, currently employed by the borough council to engage with the public on a range of health and wellbeing issues. Alternatively, the intervention may be delivered by **community workers working in partner organisations.** All people delivering the intervention will undertake the bespoke SUCCESS training. Community workers will typically have good local knowledge and existing relationships within the community.
6	HOW Describe the modes of delivery (e.g., face-to-face or by some other mechanism, such as internet or telephone) of the intervention and whether it was provided individually or in a group.	All SUCCESS sessions are delivered **one-to-one** (or in a couple/small group if requested by participants). The initial SUCCESS support session will be **face-to-face**, delivered ‘there and then’ immediately following an **opportunistic approach** by a researcher and recruitment into the study. Follow up support will be delivered as per participant’s preference (e.g. online, phone, face-to-face). If the participant has stopped smoking, they will need to meet the advisor face-to-face to give a CO reading and receive their financial incentive.
7	WHERE Describe the type(s) of location(s) where the intervention occurred, including any necessary infrastructure or relevant features.	SUCCESS will be delivered opportunistically in a variety of **community locations** including (but not limited to) social supermarkets, support groups, community cafes, colleges, workplaces.
8	WHEN and HOW MUCH Describe the number of times the intervention was delivered and over what period of time including the number of sessions, their schedule, and their duration, intensity or dose.	The SUCCESS initial support session and follow up sessions are designed to offer **brief advice** taking no longer than 20 min (although additional time for delivery will be permitted if needed). Follow up support meetings will be **offered 1 month, 2 months and 6 months** at a flexible time and location as per participant’s preference. Participants who stop smoking will be given a **£20 financial incentive at each follow up support session** providing they provide a clear CO test. A payment will be given at 6 months of an **additional £40 if they are smoke free for all follow up support sessions.**
9	TAILORING If the intervention was planned to be personalised, titrated or adapted, then describe what, why, when, and how.	SUCCESS behavioural support **is tailored to the information the participant provides** regarding smoking and vaping beliefs, motivations and goals and also the subsequent smoking and vaping behaviour following the initial support session.
10	MODIFICATIONS If the intervention was modified during the course of the study, describe the changes (what, why, when, and how).	N/A – intervention has not been implemented to date.
11	HOW WELL Planned: If intervention adherence or fidelity was assessed, describe how and by whom, and if any strategies were used to maintain or improve fidelity, describe them.	**A feasibility study** will be undertaken. This will consist of taking baseline measures from participants at recruitment and following up participants at 1 month, 3 month and 6 months post recruitment. The purpose of the baseline/follow ups will be to **test acceptability and feasibility of our proposed data collection methods** in view of a larger randomised study. **CO testing** will be used at follow up support sessions to validate quits with ≤ 8ppm. Community workers will keep a record of every attempted contact with participants and follow up rates will be recorded. **Support** (e.g. online meetings, email/phone support, What’s App group) will be offered to community workers by the research team to discuss troubleshooting. Intervention adherence will be monitored through intervention **delivery component checklists** completed post-advice session by the advisors delivering the intervention. Researchers will **conduct observations** of a sample of advice sessions, and **interview advisors and participants** following 6 month follow up. The participant interviews will not only collect data on intervention participation but will also collect perspectives on proposed data collection methods.
12	HOW WELL Actual: If intervention adherence or fidelity was assessed, describe the extent to which the intervention was delivered as planned.	N/A – intervention has not been implemented to date.

Whilst this work helped define an intervention targeting a specific seldom heard population, the findings presented may have wider relevance in light of the Government’s proposed measures to achieve the ambition of making England ‘smokefree’ by 2030 [9]. This data supports offering provision of e-cigarette starter kits to disadvantaged groups, which the Government’s ‘swap to stop’ programme hopes to deliver [35]. As part of this initiative, local authorities and SSS will potentially have flexibility to choose the mode of delivery and make decisions about which populations to target. Local authorities should consider utilising innovative community-based delivery approaches and intense combined support methods, such as those suggested in the SUCCESS model, to potentially maximise impact in the communities that need the support the most, thus reducing health inequalities.

### Strengths and limitations

The application of the TIDieR checklist as a qualitative analysis tool was effective and is of use as a framework for researchers conducting intervention development work. In addition, findings were triangulated with relevant literature, meeting notes and observations to offer a thorough exploration of optimal intervention components. For future studies, the TIDieR headings could be used to formulate topic guide questions allowing for ease of coding and reducing the need for an inductive coding stage.

The interview data was limited by the small convenience sample recruited from one deprived seaside town in the UK which may limit generalisability. However, the recruitment locations were purposefully selected to ensure a wide range of residents participating in the interviews that reflected different groups known to live in UK coastal communities (e.g. people on low incomes, older people and immigrants [8. 10]). Unfortunately, due to recruitment practices, we were not able to interview male ‘routine and manual’ workers, although we did speak to their partners and younger males training in ‘routine and manual’ professions. Participants who were included in discussions might be considered particularly ‘seldom heard’ in mainstream research, thus our embedded community approach successfully gave voice to these groups. Interviews were also relatively in depth and therefore provide a richness of insight. Participants accessing community groups showed a willingness to engage in a potential intervention which influenced the proposed community-based intervention design. However, it is possible that to reach smokers who work during the day, a flexible approach will need to be taken by community workers delivering the intervention.

As with any research exploring stigmatised behaviour, there is a risk of social desirability bias. However, we minimised this risk by reassuring participants at recruitment that they did not have to be interested in giving up smoking to take part and that they were free to voice any opinion, positive or negative, about our intervention ideas. In addition, we discussed a range of smoking cessation options with no weight given to any one approach. Relatedly, we are aware that that this study gathered views on potential intervention approaches presented hypothetically. A feasibility study, therefore, will be important in understanding the actual acceptability of the final defined intervention.

## Conclusion

People living in deprived coastal communities have some of the worst health outcomes in the UK, driven in part by high smoking rates. This population struggle to access stop smoking services and may prefer support to be delivered opportunistically within their community. Intensively combining evidence-based interventions, alongside support and encouragement from community-based stop smoking advisors, may be beneficial. Feasibility testing of the SUCCESS intervention in this population is required prior to definitive effectiveness testing. Applying the TIDieR checklist as a deductive framework for the analysis of qualitative feedback offers a systematic approach to intervention development work from conception to final design and ensures transparency of the decision-making process around inclusion of individual intervention components. Combined with triangulating other intervention development activities, analysing qualitative data in this way could result in better targeted and more effective interventions.

## Electronic supplementary material

Below is the link to the electronic supplementary material.

Supplementary Material 1

Supplementary Material 2

## Data Availability

The datasets generated and/or analysed during the current study are not publicly available due consent to participate being obtained verbally but are available from the corresponding author on reasonable request.
