# Supplementary material for: Theoretically framing views of people who smoke in understanding what might work to support smoking cessation in coastal communities: adapting the TIDieR checklist to qualitative analysis for complex intervention development
Source: BMC Public Health. 2024 Sep 9;24:2443. doi: 10.1186/s12889-024-18923-x (PMC11382369; doi:10.1186/s12889-024-18923-x)
Supplement: Supplementary file 1 — Supplementary Material 1 [file 12889_2024_18923_MOESM1_ESM.docx]

**SUCCESS study (Supporting Coastal Communities to Stop Smoking) Logic model:**

*Mechanisms of change*

ISSUE & DETERMINANTS INTERVENTION COMPONENTS INTERVENTION ACTIVITIES OUTCOMES

Context moderators

- CW time/skills/motivation to engage the community in intervention
- Level of support and promotion from local authority, community organisations and other local networks
- Cultural differences related to smoking practices

Intervention strategy

Tailored package (Intervention areas):

- Community based switching campaign
- Community workers (CW) trained to promote campaign and offer support
- Provision of e-cigarette starter kits for free
- Provision of ongoing supplies
- Monthly CO testing and ‘check in’ with CW, offering relapse prevention support
- Monthly incentives for staying smokefree
- Signposting to websites with evidence-based information about the risks of smoking, relative risks and benefits e-cigarettes as alternatives, in addition to general smoking relapse prevention information

Process measures:

**Capability**

- Increased confidence in switching
- Enhanced capacity to resist urges to smoke
- Enhanced capacity to use e-cigarettes

**Opportunity**

- Switching to e-cigarette
- Access to social support within immediate community

**Motivation**

- Evidence based beliefs about risks of smoking and e-cigarette relative risks
- Stronger sense of identity as an ex-smoker
- Reduced negative emotions/attitudes towards switching to e-cigarette
- Increase vaping acceptability and decreased tobacco acceptability

Intervention functions

- Provide alternatives / substitutes
- Promote switching, not quitting
- Community based social support (promote confidence, ‘all in it together’)
- Incentivise and reward positive behaviour change
- Education (about health risks of smoking and strategies for cessation)
- Skills training (on switching and avoiding relapse)
- Motivational (change beliefs and feelings)

BCTs

- 1.1 Goal setting (behaviour)
- 1.2 Problem solving
- 1.4 Action planning
- 1.5 Review behaviour goal(s)
- **2.6 Biofeedback (CO monitoring)**
- 3.1 Social support (unspecified)
- 4.1 Instruction on how to perform a behaviour
- 4.2 Information about antecedents
- 5.1 Information about health consequences
- 5.3 Information about social/environmental consequences
- 6.1 Demonstration of behaviour
- 7.1 Prompts/cues
- 7.7 Exposure
- 8.1 Behavioural practice/rehearsal
- **8.2 Behaviour substitution**
- 9.1 Credible source
- **10.1 Material incentive (behaviour)**
- **10.5 Social incentive**
- 11.2 Reduce negative emotions
- **12.2 Restructuring the social environment**
- 12.3 Avoidance/reducing exposure to cues for the behaviour
- 12.4 Distraction
- **13.5 Identity associated with changed behaviour**
- 15.1 Verbal persuasion about capability
- 15.3 Focus on past success

High tobacco smoking prevalence in deprived populations - coastal communities

Primary outcome:

Smoking abstinence (6 mths)

Secondary outcomes:

Smoking reduction; quality of life/health, stress, wellbeing

Capability barriers

- Lack of confidence in quitting (never tried to quit)
- Lack of confidence that abstinence can be achieved (due to previous failed quit attempts)
- Mistrust of healthcare systems
- Limited understanding of cessation support (medication, behavioural support, e-cigarettes)
- Low self-efficacy in own ability to quit
- Low levels of education

Opportunity barriers

- Cannot afford NRT or e-cigarette
- Geographical isolation
- Limited/no access to social support (e.g. friends, family, community support to quit)
- Difficult to access routine structured support to quit via SSS or GP
- Negative experiences of social support (smoking is the norm in immediate community)
- Living with a smoking partner/household smoker
- Exposure to known smoking triggers (e.g. alcohol, stress)
- Smoking as a stigmatised behaviour in wider society (becomes more inward facing towards immediate community)
- Scepticism/lack of knowledge from health professionals about ability to quit

Motivation barriers

- Lack of desire to quit
- Sense of hopelessness towards positive health behaviour change
- Belief that smoking is a ‘treat’ or reward
- Belief that NRT or e-cigarettes continues undesired dependence (e.g “not really giving up”)
- Concerns about health risks of e-cigarettes
- Concerns about health risks of nicotine
- Concerns about safety of devices
- Rejection of a non-smoking and/or vaping identity
- Belief social support would not be helpful
