# Supplementary material for: Theoretically framing views of people who smoke in understanding what might work to support smoking cessation in coastal communities: adapting the TIDieR checklist to qualitative analysis for complex intervention development
Source: BMC Public Health. 2024 Sep 9;24:2443. doi: 10.1186/s12889-024-18923-x (PMC11382369; doi:10.1186/s12889-024-18923-x)
Supplement: Supplementary file 2 — Supplementary Material 2 [file 12889_2024_18923_MOESM2_ESM.docx]

**SUCCESS Study Discussion guide**

**Demographics**

Can I just start by asking you a few questions about yourself?

Age

Ethnicity

Postcode

Smoking Status (Smoker, Vaper, Dual user, other)

Time since quit

Quit method used

Age started smoking

Employment status

Occupation

Home ownership

**Topic guide**

**Warm Up - Smoking history**

- Can you start by telling me a bit about when you started smoking and what was happening around that time?
- How long have you been smoking for? *Prompts: What do you smoke? How many a day?*
- Have you ever tried to quit? *Prompts: What helped/hindered you?*
- **Smokers** - How do you feel about your smoking? Are you looking to quit? What do you think would help you? Why might you want to quit?
- **Recent quitters** – What led to your recent quit? How were you feeling about your smoking at that time? How do you feel about smoking now? How do you feel about other people smoking now you have quit? *Prompts:* *What support did you find helpful? E.g. SSS, E-Cig, NRT, peer support, health scare, other*

**Community**

- What does community mean to you? *Prompts: Who do you consider to be part of your community? Prompts: Family/Friends/ Work etc.*
- What is it like living by the sea? *Prompts: Do you go to the beach? What is like having tourists come here for the Summer? Does it feel different living here in Summer? Does it impact your smoking in any way?*
- *Do you think where you live influences your smoking?*
- Where do you see smoking/vaping in this area?
- What are the people like who smoke/vape? Is there any difference between the people who smoke and those who vape?

**Our ideas/Your ideas:**

**E-Cigarettes**

One idea we’re considering is giving e-cigarettes to people to help them to quit.

- What do think about e-cigarettes and vaping? *Prompts: If impressions are negative – The science tells us that e-cigarettes are much safer than smoking. What do you think would change your mind about using them?*
- Have you ever used an e-cigarette? *Prompts: Types, preferences, cost, convenience.*
- Where would you like to be given the e-cigarette? And by who? Probe for GP, SSS, workplace, event, food bank, delivered by post etc.
- Would you prefer to be given an e-cigarette or given a voucher to buy one from a shop?
- Do you this approach would help people and why?
- How would you feel about being sent further supplies by post?

**Behavioural Support**

Another idea was to offer people stop smoking support. This could involve attending a group or could be provided one-to one or connecting with people on social media.

- What do you think about this idea? Have you used the local stop smoking service and received support in the past? *Prompts: How did you find that support?*
- *Did you know that you are three more times more likely to quit with some behavioural support? Does this change your view?*
- What would be the best place to receive support – go somewhere/ someone come to your workplace/ online via a videocall/ via a phone call/ someone come to a community location e.g. community centre or food bank/someone come to your home/remote sessions? Website/apps/Social media/what app’s groups?
- Do you this approach would help people and why?

**Financial incentive**

We are also considering giving people money to give up smoking, as evidenced by a urine test.

- What do you think about this idea?
- How much do you think would be suitable? When should people get the payment and in what format? Probe for SSS sessions, community outreach, bank account, vouchers
- What about some payment going towards something for your community? For example, play equipment, a community event. Do you have any ideas?
- Do you this approach would help people and why?
- Would people mind providing a urine sample?
- We thought we would follow up people on a monthly basis for 6 months. What do you think about this idea?

**Other**

We thought about offering all three things at once? What do you think about this? *Prompts: Can you think of any reasons why this would or would not help people?*

- How do you think we should publicise the intervention? Probe for local media and social media, word of mouth, community groups etc.
- **YOUR IDEAS:** We really want to know **what you think** would help people to quit smoking. Other things that have been tried include text message reminders and apps. What are your thoughts? *Prompts: What is the best place to support people? Who are the people that should deliver it? E.g. community leaders or stop smoking advisor. How would you feel about being approached to take part at a food bank (tailor to relevant location for participant)*

**Recruitment**

- How would be the best way to recruit people to take part in this research?
- How would you feel about randomisation (provide an explanation)?
- Why might people be reluctant to take part?
- How would you like to be followed up? Text? Post? Phone-call?

Anything else? Thank you.
